# Investigating impact of cardiorespiratory fitness in reducing brain tissue loss caused by ageing

**DOI:** 10.1093/braincomms/fcab228

**Published:** 2021-11-18

**Authors:** Shinjini Kundu, Haiqing Huang, Kirk I Erickson, Edward McAuley, Arthur F Kramer, Gustavo K Rohde

**Affiliations:** 1 Medical Scientist Training Program, University of Pittsburgh, Pittsburgh, PA 15261, USA; 2 Brain Aging & Cognitive Health Lab, Department of Psychology, University of Pittsburgh, Pittsburgh, PA 15260, USA; 3 Department of Kinesiology and Community Health, University of Illinois Urbana-Champaign, Champaign, IL 61801, USA; 4 Beckman Institute, University of Illinois at Urbana-Champaign, Urbana, IL 61801, USA; 5 Department of Psychology, Northeastern University, Boston, MA 02115, USA; 6 Biomedical Engineering, Electrical and Computer Engineering, University of Virginia, Charlottesville, VA 29908, USA

**Keywords:** cardiorespiratory fitness, brain, ageing, structural magnetic resonance imaging, transport-based morphometry

## Abstract

Mitigating the loss of brain tissue due to age is a major problem for an ageing population. Improving cardiorespiratory fitness has been suggested as a possible strategy, but the influenceon brain morphology has not been fully characterized. To investigate the dependent shifts in brain tissue distribution as a function of cardiorespiratory fitness, we used a 3D transport-based morphometry approach. In this study of 172 inactive older adults aged 58–81 (66.5 ± 5.7) years, cardiorespiratory fitness was determined by *V*$O_{2}$ peak (ml/kg/min) during graded exercise and brain morphology was assessed through structural magnetic resonance imaging. After correcting for covariates including age (in the fitness model), gender and level of education, we compared dependent tissue shifts with age to those due to $VO_{2}\;\mathrm{peak}$. We found a significant association between cardiorespiratory fitness and brain tissue distribution (white matter, *r* = 0.30, *P* = 0.003; grey matter, *r* = 0.40, *P* < 0.001) facilitated by direct visualization of the brain tissue shifts due to cardiorespiratory fitness through inverse transformation—a key capability of 3D transport-based morphometry. A strong statistical correlation was found between brain tissue changes related to ageing and those associated with lower cardiorespiratory fitness (white matter, *r* = 0.62, *P* < 0.001; grey matter, *r* = 0.74, *P* < 0.001). In both cases, frontotemporal regions shifted the most while basal ganglia shifted the least. Our results highlight the importance of cardiorespiratory fitness in maintaining brain health later in life. Furthermore, this work demonstrates 3D transport-based morphometry as a novel neuroinformatic technology that may aid assessment of therapeutic approaches for brain ageing and neurodegenerative diseases.

## Teaser

Cardiorespiratory fitness is a potential strategy to reduce brain tissue loss caused by ageing. Kundu et al. report a statistically important benefit of cardiorespiratory fitness on brain morphology using a novel automated discovery and visualization technique called 3D transport-based morphometry—prompting new questions about cardiorespiratory fitness in brain ageing and dementia.

## Graphical Abstract

**Graphical Abstract:**
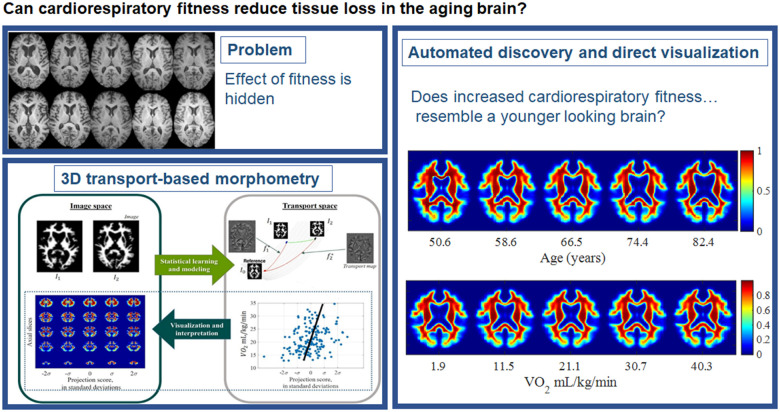

## Introduction

Late adulthood is marked by a host of physical changes and brain atrophy is one of the most ubiquitous. Specifically, after the age of forty, brain volume declines at a rate of about 5% per decade.^1^^,^^2^ Furthermore, ageing-related shifts in brain morphology are associated with concomitant declines in cognitive performance.^3^ As our population ages, there is paramount interest in strategies to potentially mitigate the brain tissue loss that occurs with ageing. In recent research, cardiorespiratory fitness (CRF) has been described to be neuroprotective in older adults.^4–10^ As CRF can be influenced through exercise intervention, there may be future potential for these therapies in mitigating neurodegeneration.

However, the influence of CRF on brain tissue has not been fully characterized quantitatively. Tissue atrophies in the ageing brain non-uniformly across multiple regions.^11^ Multiple studies have demonstrated that both ageing and decreased CRF are associated with non-uniform declines.^5^^,^^12^ Yet, prior studies investigating associations with CRF have not characterized differential atrophy and degeneration across the brain. First, conventional statistical methods comparing regional volumes^10^ and voxelwise metrics^11^^,^^13^^,^^14^ are insufficiently sensitive to the spatial interdependence in brain tissue, and its nonlinearity. Indeed, regional volumes have led to varying reports of the degree to which tissue shifts dependent on age and those dependent on CRF overlap.^14^^,^^15^ In contrast, new techniques that measure spatial variation in brain tissue as mathematical distributions can directly measure these diffuse, non-linear processes. Second, while regional volumes and voxelwise metrics are basic statistical descriptors, they do not correspond to any biophysical properties of brain tissue. In other words, these descriptors cannot be used to construct a visualizable brain phenotype for interpretation—that is, they are not *generative.* Third, previous approaches required numerical descriptors (i.e. region-of-interest volumes, voxelwise statistics, etc.) to be specified by the user *a priori*. However, automated pattern analysis can enable the discovery of complex phenomena that numerical descriptors cannot capture and may allow for expanded analysis.

In recent work, the authors developed an automated approach to discover discriminant phenotypic patterns from brain images by directly measuring the spatial tissue distribution. This approach enabled biophysical properties of the brain to be modelled as mass transport, and also yielded a *generative* approach. The technique is called 3D transport-based morphometry (TBM).^16^ This paper applies the novel TBM approach to extract the perturbations in brain phenotype statistically explainable by CRF. TBM quantifies the effort required to morph one image into another by using the mathematics of optimal mass transport^16^ to enable discovery of phenotypic patterns using machine intelligence, in contrast to conventional methods comparing similarity through statistical descriptors. Finally, because it is generative, models constructed using TBM may be interrogated using inverse transformation to yield immediately visualizable brain images illustrating the biophysical features that cause the relationship with CRF.

The goal of this research is to discover and visualize the shifts in brain tissue distribution that are most strongly associated with CRF in an automated manner using the TBM technique. Furthermore, this study aims to determine the degree to which the pattern of tissue distribution with higher CRF overlaps with the distribution of ageing-related losses. We hypothesize that lower CRF would be associated with altered tissue distribution in a regionally specific fashion, disproportionately associated with the frontal and temporal regions to closely mirror those changes that undergo characteristic changes in ageing. The following are the contributions of this paper:

- We describe a framework for automated discovery and visualization of latent patterns in brain tissue distribution using 3D TBM.
- We validate that 3D TBM can uncover characteristic ageing-related phenotypic changes in an automated manner.
- We discover the pattern of brain tissue distribution most strongly associated with CRF using TBM.
- We directly visualize the characteristic phenotypic shift most associated with CRF in white matter and grey matter through inverse TBM transformation.
- We determine the degree of statistical overlap between the dependent phenotypic shifts due to ageing and CRF.

## Materials and methods

### Participant characteristics

**Figure 1:**
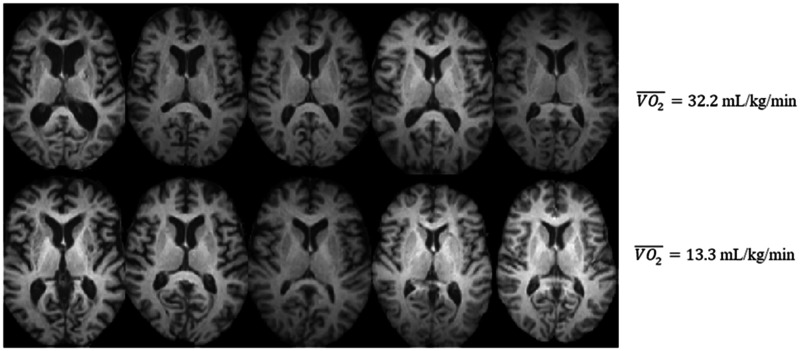
**Brain images of older adults. High-resolution structural images belonging to 10 individuals.** The same axial slice is displayed for all subjects. The top row illustrates the top 5 most fit individuals in the dataset (average $\bar{VO_{2}}$ = 32.2 ml/kg/min) and the bottom row illustrates the 5 least fit individuals in the dataset (average $\bar{VO_{2}}$ = 13.3 ml/kg/min).

In this study, 172 healthy community-dwelling older adult subjects were recruited at the University of Illinois. Informed consent was obtained from all participants. These subjects were enrolled as part of a randomized controlled exercise trial. This study focused on a cross-sectional analysis of their baseline data to assess the relationship between CRF and brain tissue distribution. Subject demographics are summarized in Table 1. Sample structural images are shown in Fig. 1. There was no significant difference in the range of ages across the male and female participants of this study (*P* = 0.75). Results on a small subset of these patients were previously published by (16). The purpose of the earlier work was to validate the approach utilized in this paper, whereas the current study assesses the effects of CRF on the brain.

**Table 1:** Subject demographics

Age (years)	58–81 (66.5 ± 5.7)
Gender (M:F)	60:112
Education level	2–8 (1 = 0%; 2 = 0.6%; 3 = 0.6%; 4 = 18.6%; 5 = 28.5%; 6 = 19.2%; 7 = 22.1%; 8 = 10.5%)
Body mass index (kg/m^2^)	18.9–42.6 (28.9 ± 4.5)
Total brain parenchymal volume (L)	0.75–1.7 (1.2 ± 0.2)

There was no significant difference in the range of ages across the male and female participants of this study (*P* = 0.75). Education levels: 1 = less than 7th grade; 2 = 9th grade (junior high); 3 = partial high school; 4 = high school graduate; 5 = 1–3 years of college or 2 year college; 6 = college/university degree; 7 = master's degree; 8 = PhD or equivalent. Study population demographics are reported as a range, followed by mean ± standard deviation.

Inclusion criteria were as follows: age between 55 and 80 years, being physically inactive as defined by engaging in ≤30 min of exercise each week within 6 months prior to baseline examination,^17^ demonstrating strong right handedness determined by score of ≥75% on the Edinburgh Handedness Questionnaire,^18^ scoring ≥51 on modified Mini Mental Status Examination^19^ to rule out clinical cognitive impairment, normal colour vision and visual acuity of at least 20/40, and no history of neuropsychiatric conditions or neurological diseases, including Parkinson’s disease, multiple sclerosis, Alzheimer’s disease, stroke, or infarcts.

Exclusion criteria were as follows: score greater than 3 on the Geriatric Depression Scale that could indicate possible depression,^20^ history of cardiovascular disease, evidence of chronic inflammation (i.e. severe arthritis, psoriasis, inflammatory bowel disease, asthma, polyneuropathies, Lupus) and metal implants as contraindications for MRI.

### MRI acquisition

High-resolution T_1_-weighted images of the brain were collected on a 3 T head-only Siemens Allegra MRI scanner at the University of Illinois. Images were acquired using a 3D MPRAGE (Magnetization Prepared Rapid Gradient Echo Imaging) protocol in which 144 contiguous axial slices collected in ascending fashion parallel to anterior posterior commissures. Scan parameters were as follows: echo time = 3.87 ms, repetition time = 1800 ms, field of view = 256 mm, acquisition matrix 192 × 192, slice thickness = 1.3 mm, flip angle = 8°.^21^

### Cardiorespiratory fitness assessment

CRF ($VO_{2}$_peak_) was assessed by graded maximal exercise testing on a motor-driven treadmill. Participants were required to obtain consent from their personal physician before CRF testing was conducted. The participant walked at a speed slightly faster than their normal walking pace (approximately 30–100 m/min or 1.8–6 km/h) with increasing grade increments of 2% every 2 min. A cardiologist and nurse continuously monitored measurements of oxygen uptake heart rate and blood pressure. Resting heart rate was measured while the participant lay in the supine position after ECG preparation, and before the treadmill test began. Oxygen uptake ($VO_{2}$) was measured from expired air samples taken at 30-s intervals until a maximal $VO_{2}$ was attained or to the point of test termination due to symptom limitation and/or volitional exhaustion. $VO_{2}$ peak was defined as the highest recorded $VO_{2}$ value when two of three criteria were satisfied: (i) a plateau in $VO_{2}$ peak between two or more workloads; (ii) a respiratory exchange ratio > 1.00; and (iii) a heart rate equivalent to their age predicted maximum (i.e. 220—age).^17^ The $VO_{2}$ peak was corrected for participant body weight. Study participant exercise variables are summarized in Table 2.

**Table 2:** Exercise variables

Resting systolic blood pressure (mmHg)	100–180 (134 ± 15)
Resting heart rate (bpm)^a^	49–120 (73 ± 13)
Resting diastolic blood pressure (mmHg)	54–104 (80 ± 8)
Peak systolic blood pressure (mmHg)	132–236 (182 ± 21)
Peak heart rate (bpm)	91–234 (158 ± 20)
Peak diastolic blood pressure (mmHg)	58–110 (86 ± 10)
VO_2_ rate (L/min)	0.6–3.2 (1.7 ± 0.5)
VO_2_ peak (ml/kg/min)	12.9–34.7 (21.1 ± 4.7)

a Resting heart rate was missing for one subject. Study population exercise parameters are reported as a range, followed by mean ± standard deviation.

### Registration, segmentation and image pre-processing

The brain images were all co-registered from native space to the Montreal Neurological Institute space using a 12-parameter affine transformation. Subsequently, in the pre-processing phase, images were skull-stripped and segmented into grey matter (GM) and white matter (WM) component images for all subjects using the Statistical Parametric Mapping^22^ software version 12. After segmentation, the respective WM and GM images were smoothed using a 3D Gaussian filter with full-width-at-half-maximum 2.35. For subsequent transport-based analysis, high-resolution structural images were normalized to have equal total mass. Study population references for GM and WM images were generated by taking the Euclidean mean of respective GM and WM images, respectively. Therefore, the images are normalized such that $\int I_{0}\left(x\right)dx=\int I_{i}\left(x\right)dx=\;1,$ where $I_{0}\left(x\right)$ is a common reference for all i={1,…,N} images in the study population, where *N* is the number of subjects. The purpose of the reference images was to enable the metric space in the transport domain to be defined,^16^ which is further described in Automated pattern analysis using 3D TBM. Anatomic localization was performed using the Montreal Neurological Institute automated anatomic labelling atlas, whose details are in Localizing regions of dependent tissue displacement.

### Automated pattern analysis using 3D transport-based morphometry

3D TBM measures the structural similarity among brain images by quantifying the mass distribution of brain tissue. Non-uniform atrophy and degeneration modifies the relative spatial distribution of tissue across regions (i.e. lower relative tissue density in areas of atrophy, higher relative tissue density in areas of preservation). We quantify these structural perturbations using the mathematics of optimal mass transport^23^ by measuring relative shifts in tissue spatial distribution. Mathematically, tissue spatial distribution is quantified using the using the L2-Wasserstein distance, a nonlinear metric with theoretical foundations and mathematical guarantees further described in Supplementary material A3.^24–27^

As 3D TBM focuses on mass distribution of signal intensity, for each source brain image $I_{i}(x)$, 3D TBM generates a mapping for each brain image called a *transport map*, which quantifies how to rearrange the source image to result in template image $I_{0}(x)$, with the constraint that the mass moved must be minimized. TBM can be used to *transform* the image from its *image domain* representation $I_{i}(x)$ to its *transport domain* representation $f_{i}^{*}\left(x\right)$ using the TBM equation for analysis.

(1) $$f^{*}\left(x\right)=\;\underset{f\in \mathrm{MP}}{\mathrm{arg min}}\int {\left(f\left(x\right)-x\right)}^{2}I_{0}\left(x\right)dx,$$

such that $\det\left(Df\left(x\right)\right)I_{1}\left(f\left(x\right)\right)=I_{0}(x)$

**Figure 2:**
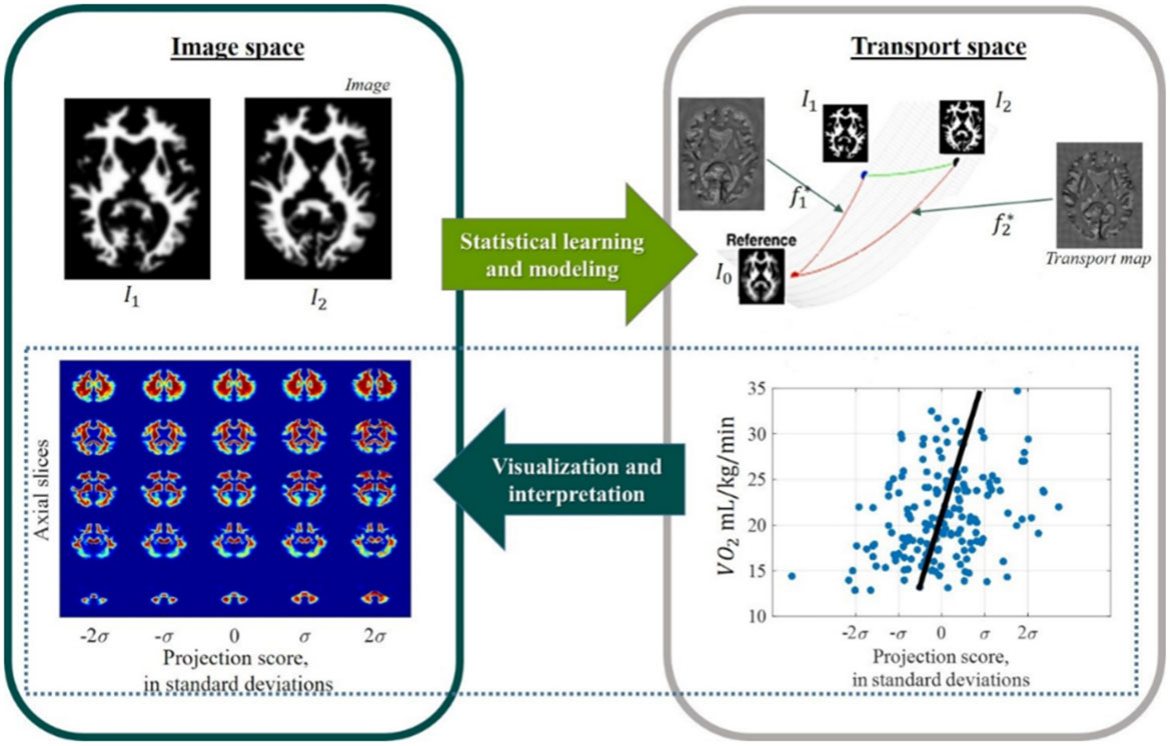
**Transport-based morphometry system diagram.** Images are mapped to corresponding transport maps through a unique, one-to-one transformation using optimal mass transport to facilitate statistical learning and modelling. Modelling functions computed in transport space can be inverted to visualize and interpret the corresponding images.

Here, $f^{*}\left(x\right)$ is a transport map corresponding to image $I_{i}(x)$. MP defines the space of all mass-preserving mappings, and *D* corresponds to the Jacobian operator. The transport map is a vector field describing how to morph the brain tissue distribution into the common reference, capturing complex variations in both shape and texture. The system diagram is shown in Fig. 2. GM and WM tissues were analysed separately in this study.

The mapping from image domain to transport domain through (1) is a bijective and invertible transformation. In other words, under a set of constraints that TBM satisfies, each image corresponds to a unique transport map and vice versa, illustrated in Fig. 2. Furthermore, the underlying structure of the metric space in the transport domain is a Riemannian manifold,^16^ where samples in the dataset comprise points on the manifold; this enables the key property of TBM—that it is *generative* (see Supplementary material A3). A new point in the transport domain can be interrogated to generate direct visualization of the brain image with phenotypic change.^16^ Mathematically, a given transport map can be inverted to visualize the corresponding brain image according to the synthesis equation.

(2) $$I\left(x\right)=\det\left(Df^{-1}\left(x\right)\right)I_{0}\left(f^{-1}\left(x\right)\right),$$

where $f^{-1}\left(x\right)$ is the inverse mapping of $f\left(x\right)$.

Here, $I\left(x\right)$ is a computer-generated brain image illustrating the tissue displacements captured by a given point in the transport space. Equation (2) enables the key advance of TBM, which is direct visualization of brain images illustrating the biophysical perturbations represented by statistical models in the transport domain, which makes TBM *generative*.

Brain tissue distribution captures information not assessed through volume measures alone (Supplementary Fig. A1) and better characterizes the underlying structural variation^16^ compared to deformation-based measures such as in voxel-based morphometry^13^ and the original image domain (Supplementary material A4). Unlike numerical descriptors in traditional methods which are often defined on an *ad hoc* basis, 3D TBM is based on a physical model of joint spatial image intensities, with intensity being proportional to the probability of observing a particular tissue type at that location. The interested reader is referred to Kundu et al.^16^ for further measure-theoretic description.

### Statistical analysis: Assessing statistical relationship with clinical variables

Transport-maps were vectorized and concatenated as columns of a standard data matrix $D\in R^{p\times n}$ for subjects 1…n, where p is the number of elements in each transport map. Principal components analysis (PCA) technique was used to remove the data dimensions with little or no contribution to the overall data variance before computing the most correlated direction with CRF according to the following equation:

(3) $$D=V\Lambda U^{T}=VX.$$

**Figure 3:**
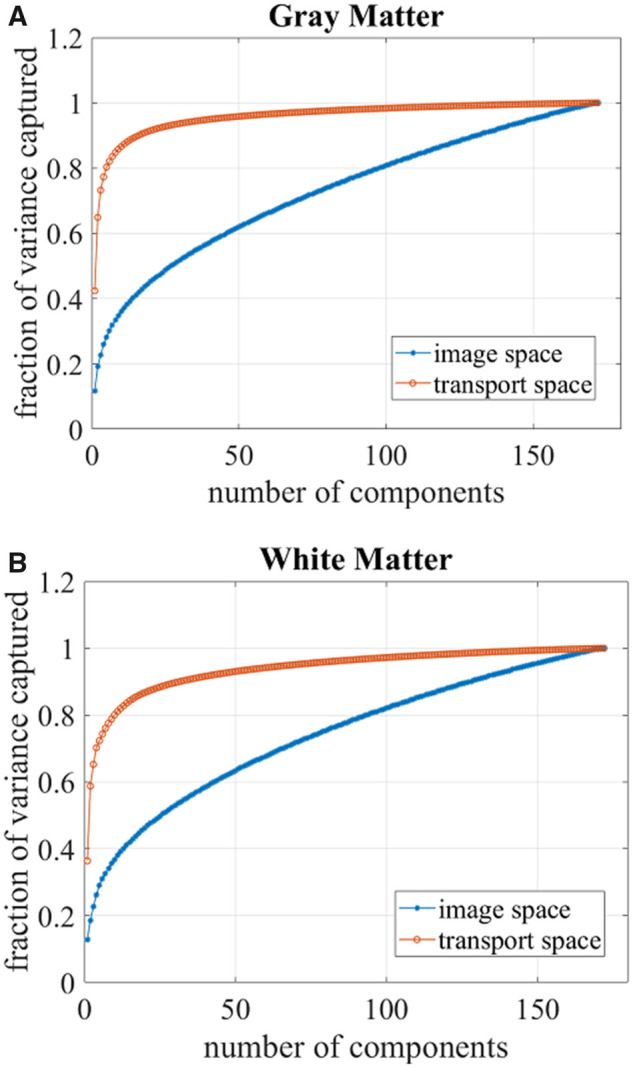
**Variance plots.** Variance plots showing number of principal components needed to capture the variation in the data for (**A**) grey matter tissue and (**B**) white matter tissue. Fewer components are needed to capture the variance in the transport space compared to the image space, suggesting that the transport domain features better capture structure in the data.

Here, the columns of matrix $V\in R^{p\times \left(n-1\right)}$ contain the n-1 eigenvectors that span the space in which the transport maps lie. According to (3), all the information in the data matrix D can be summarized by the n-1 orthonormal eigenvectors. The data matrix is projected onto the *d* topmost eigenvectors associated with 90% of the variance in the dataset, approximating the inflection of the TBM variance plots in Fig. 3A and B. Thus, we create the reduced-dimension, centred data matrix $X\in R^{d\times n}$.

The direction in transport domain that best describes the common tissue shifts associated with an independent variable $v\in R^{n\times 1}$ is computed according to the following equation^16^^,^^24^:

(4) wcorr=arg maxw⁡wTXvwTw=XvvTXTXv.

Here, $w_{\mathrm{corr}}$ is a vector field that summarizes the direction and magnitude by which tissue is displaced as a function of v. The field $w_{\mathrm{corr}}$ is calculated as a composite from all brain images and summarizes the specific phenotypic shift with respect to the common reference most explained by linear correlation with independent variable v. *De novo* images that visualize the influence of $w_{\mathrm{corr}}$ are calculated through inverse transformation from the transport map generated from *w* in (5). Here, $\overset{–}{x}$ refers to the mean transport map across the study population and *t* represents the increment or decrement of samples along the computed direction.

(5) $$w=\overset{–}{x}+tw_{\mathrm{corr}}.$$

The main effects of ageing on GM and WM were assessed by computing the direction in the transport domain exhibiting strongest linear correlation with age. Let $y\in R^{n\times 1}$ be the column vector representing the ages for the subjects in the study. The most correlated direction in transport space is computed by setting v=y in (4).

#### Assessing brain outcomes associated with cardiorespiratory fitness

We also assessed the brain tissue outcomes in relation to CRF. In this case, y is set as the $VO_{2}$ peak. The influence of confounding variables of age, gender, and level of education were removed before performing the regression analysis using the formula in the following equation:

(6) $$v=y-Z{\left(Z^{T}Z\right)}^{-1}Z^{T}y,$$

where $Z\in R^{n\times c}$ is the matrix containing c covariates. The variable v represents the component of y that is uncorrelated and orthogonal to the confounding variables in Z. The analyses were performed separately for GM and WM tissues. Given that education was reported as a potential factor to mitigate age-related changes, we included the latter as a covariate in our model.^11^

#### Null hypothesis testing

Statistical significance of the computed directions $w_{\mathrm{corr}}$ were assessed by permutation testing with *T* = 10000 tests to determine what fraction of the time a stronger correlation could be achieved when labels and transport maps are randomly assigned.

#### Assessing interaction between age and cardiorespiratory fitness

We assessed the degree to which the tissue shifts associated with increasing age and lower CRF overlap. We calculated the linear dependence between brain tissue displacements as functions of age and $VO_{2}$ peak, respectively. Pearson’s correlation coefficient was calculated between the respective directions computed in the transport space (tissue displacement versus age and tissue displacement versus $VO_{2}$ peak). GM and WM tissues were assessed separately. Statistical significance was assessed by permutation testing with *T* = 10000 tests.

Next, we studied whether higher CRF could be an effect modifier on the relationship between decreasing age and brain tissue distribution. The effect of age x CRF was assessed by performing regression of brain tissue distribution in the transport space setting $v=(\mathrm{age}-\bar{\mathrm{age}})(VO_{2}\;\mathrm{peak}-\bar{VO_{2}\;\mathrm{peak}})$, where the mean-subtracted age and $VO_{2}\;\mathrm{peak}$ were multiplied to test whether CRF significantly modifies the effect of age on brain tissue distribution. Covariates of gender and level of education were included in the model and the partial effect of age was removed from CRF.

### Localizing regions of dependent tissue displacement

The pattern of dependent tissue displacements with age and with $VO_{2}$ peak was localized to specific regions of interest in the brain. The pattern of tissue displacement with $VO_{2}$ peak was corrected for covariation in age, gender, and level of education. The automated anatomic labelling atlas was used in the Montreal Neurological Institute space as a reference, with 116 anatomic regions segmented as described in Tzourio-Mazoyer et al.^28^ Of these regions, we selected the 90 regions segmenting the cerebral cortex. The T1-weighted reference image $I_{0}$ was first registered to MNI152 T1 template using FSL FLIRT (FMRIB’s Linear Image Registration Tool) and was further refined using FSL FNIRT (FMRIB’s Non-linear Image Registration Tool). The affine transformation matrix of linear transformation and non-linear warp coefficients from non-linear mapping were generated, and the inverse transformations were computed. The inverse transformations were applied on automated anatomic labelling template to transform the ROIs into reference image space.

The computer-generated image demonstrating characteristic brain tissue distribution due to a 10-year increase in age was obtained through inverse TBM transformation. Similarly, the computer-generated image demonstrating the effects of a 10 ml/kg/min decrease in $VO_{2}$ peak was also produced through inverse TBM transformation. The fractional change in tissue density per voxel with respect to the mean image was computed for each of the 116 regions for both characteristic images.

A table was generated displaying the mean fractional change in tissue density per region due to the 10-year increase in age and due to the 10 ml/kg/min decrease in CRF. In particular, the fractional change in tissue density with increasing age and with lower CRF were compared for linear dependence. The regions exhibiting a statistically significant linear correlation between age-dependent fractional density changes and CRF-dependent fractional density changes were identified through permutation testing with *T* = 10000 tests. The Bonferroni correction was applied to counteract the effect of multiple comparisons. The family-wise error rate was set at <0.05.

We implemented all statistical analysis codes in MATLAB (MathWorks, Natick, MA).

### Data availability

Data and code may be available upon reasonable request.

## Results

### Principal component phenotypes

The transport domain representation for both WM and GM tissues required fewer principal components to capture the same fraction of the variance when compared to the image domain representation. As Fig. 3 illustrates, the transport approach better characterizes the structure of the data when compared to the image space representation of images as pixels on a fixed grid. In the transport domain, 90% of the variance is described by 16 GM components and 32 WM components. In the image domain, 90% of the variance is described by 131 GM components and 128 WM components. Therefore, representing GM and WM tissues in terms of the spatial tissue distribution in the transport domain enables a sparser representation of the data.

### Age-related brain tissue

#### Age-related grey matter tissue

**Figure 4:**
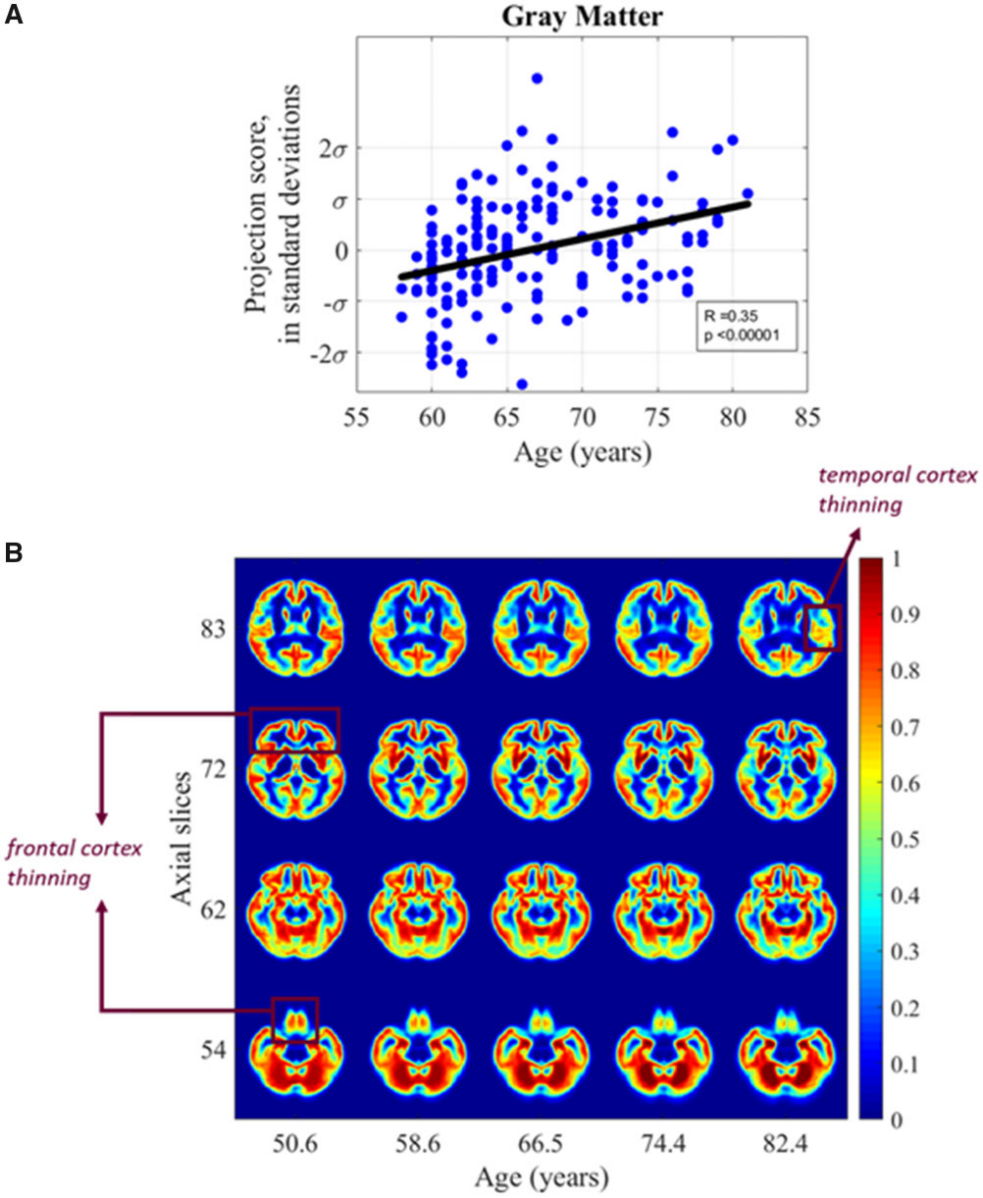
**Grey matter versus age.** (**A**) Scatter plot showing relationship between subject age and grey matter distribution in the study cohort (**B**) synthetic images generated by transport-based morphometry directly visualizing the shifts in grey matter distribution with age extrapolated from the study population. Voxels have been colorized for ease of interpretation. Select images in the *z*-plane are shown that best summarize the differences. Scale bars correspond to the normalized intensity for each generated 3D image.

TBM identifies a significant positive correlation between GM tissue distribution and age when assessed in the transport domain (Pearson’s *r* = 0.35, *P* < 0.001). Figure 4A illustrates the dependent shifts in GM distribution between GM and age when the images are projected onto the most correlated direction. The computed direction in Fig. 4A is then inverted using inverse TBM transformation to visualize the dependent shifts in brain tissue distribution that occur as a function of age. The images in Fig. 4B are computer-generated by inverse TBM transformation to visualize the shifts discovered through automated TBM analysis. Tissue shifts across varying ages were extrapolated based on the generative TBM model. Figure 4B shows selected axial slices of brain images generated by sampling along the line of best fit. The axial slices that best summarize the differences are shown, with the mean image in the dataset represented by projection score zero. The changes correspond to an average 0.78 mm displacement in GM tissue every 10 years. With increasing age, the density of the cortex appears to thin. Interestingly, there is visual thinning seen in the frontal lobes, as evident in slices 54, 62 and 72 of Fig. 4B. There is marked temporal thinning as well in slice 54 and slice 72 compared to other areas of the brain.

#### Age-related white matter tissue phenotype

**Figure 5:**
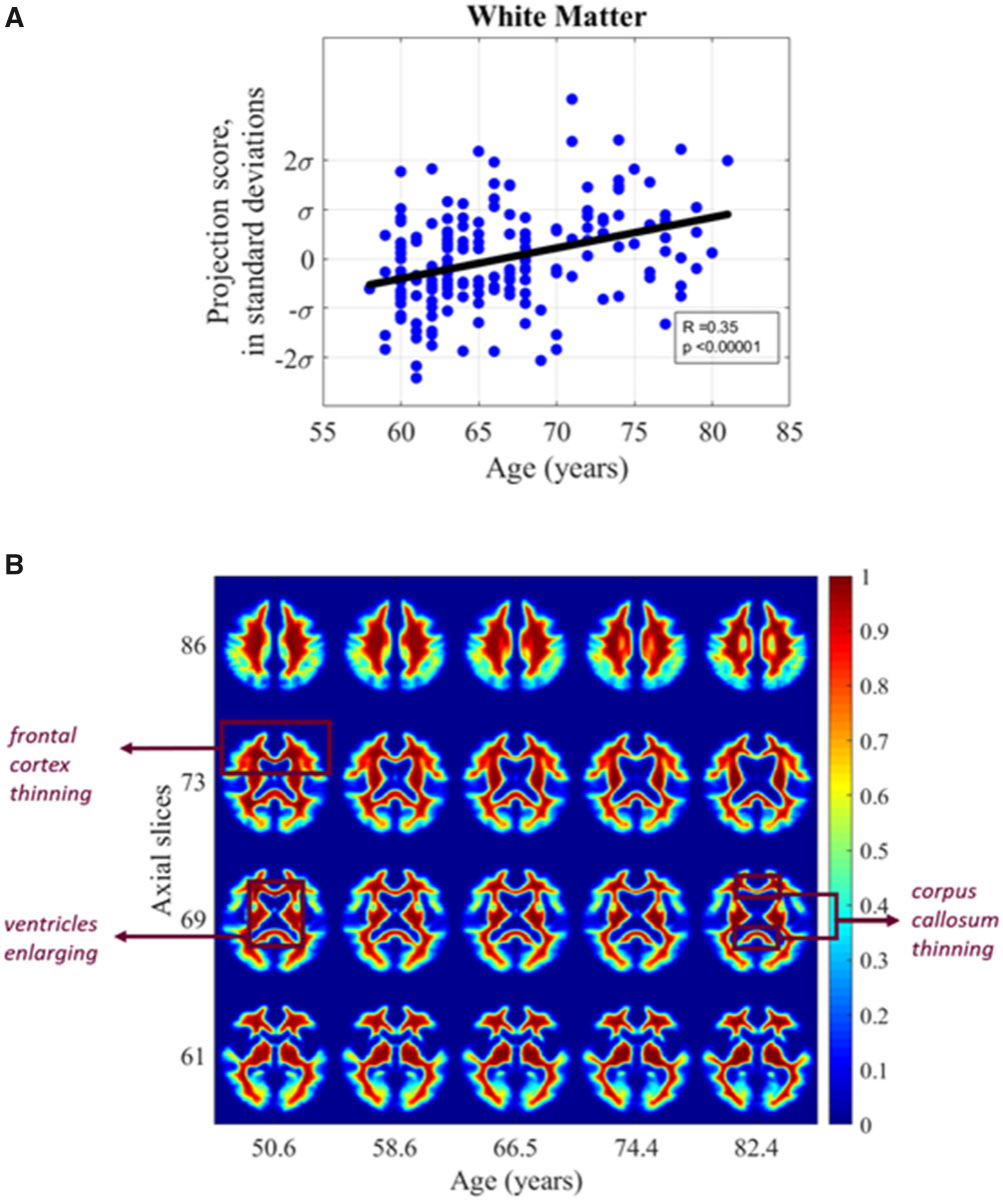
**White matter versus age.** (**A**) Scatter plot showing relationship between subject age and white matter distribution in the study cohort (**B**) synthetic images generated by transport-based morphometry directly visualizing the shifts in white matter distribution with age extrapolated from the study population. Voxels have been colorized for ease of interpretation. Select images in the *z*-plane are shown that best summarize the differences. Scale bars correspond to the normalized intensity for each generated 3D image.

We found a significant positive correlation between WM tissue distribution and age when assessed in the transport domain (Pearson’s *r* = 0.35, *P* < 0.001). Figure 5A illustrates the images when projected onto the most correlated direction. The *de novo* images in Fig. 5B are computer-generated by inverse TBM transformation to visualize the shifts discovered through automated TBM analysis. These images illustrate the changes in WM distribution associated with advancing age when inverse TBM transformation is used to visualize selected axial slices of brain images generated by sampling along the line of best fit by varying *t* in (5). Tissue shifts across varying ages were extrapolated based on the generative TBM model.

Viewing the WM tissue density in Fig. 5B, we see that as age increases, there is marked enlargement in the ventricle size. In addition, as particularly evident in slice 73, it is interesting to see a visual loss of WM density from the frontal lobes compared to other regions of the brain, described further in Localizing regions of dependent tissue displacement. In slice 69, there is loss of tissue mass from the corpus callosum. The shifts correspond to an average 0.79 mm displacement in WM tissue every 10 years.

### Association of cardiorespiratory fitness with brain tissue

#### Cardiorespiratory fitness and grey matter

**Figure 6:**
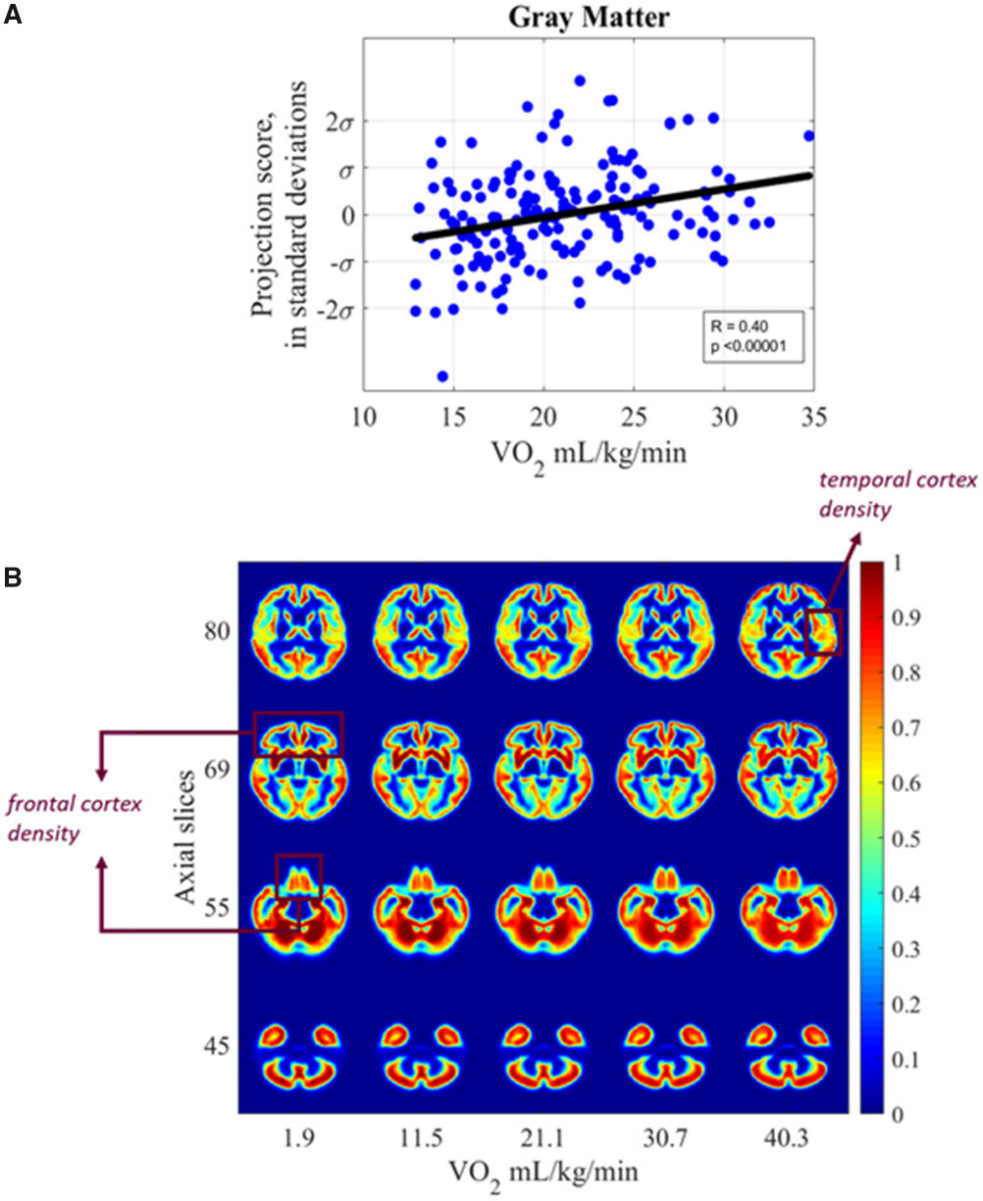
**Grey matter versus cardiorespiratory fitness.** (**A**) Scatter plot showing relationship between subject $VO_{2}$ peak and grey matter distribution in the study cohort (**B**) synthetic images generated by transport-based morphometry directly visualizing the shifts in grey matter distribution with $VO_{2}$ peak extrapolated from the study population. Voxels have been colorized for ease of interpretation. The images have been colorized for ease of interpretation. Scale bars correspond to the normalized intensity for each generated 3D image. Select interpolated images in the *z*-plane are shown that best summarize the differences.

The partial correlation between GM distribution and CRF in $VO_{2}$ peak was found to be statistically significant after controlling for covariates of age, gender, and level of education (Pearson’s *r* = 0.40 and *P* < 0.001) (Fig. 6A). The images corresponding to the statistical relationship in transport domain are shown in Fig. 6B. The images in Fig. 6B are computer-generated by inverse TBM transformation to visualize the shifts discovered through automated TBM analysis. Tissue shifts across varying CRF levels were extrapolated based on the generative TBM model. Examining the differences in tissue distribution from lower to higher CRF after correcting for covariates, several associations are evident. Notably, the shifts in tissue distribution with higher fitness levels overlap with those seen in ageing with opposite directionality, described further in Localizing regions of dependent tissue displacement. For example, we observe greater density of GM as a function of CRF preferentially in the frontal and temporal areas as illustrated most prominently by slices 55 and 69. The differences correspond to an average 0.73 mm displacement in GM tissue for every 10 ml/kg/min decrease in CRF.

#### Cardiorespiratory fitness and white matter

**Figure 7:**
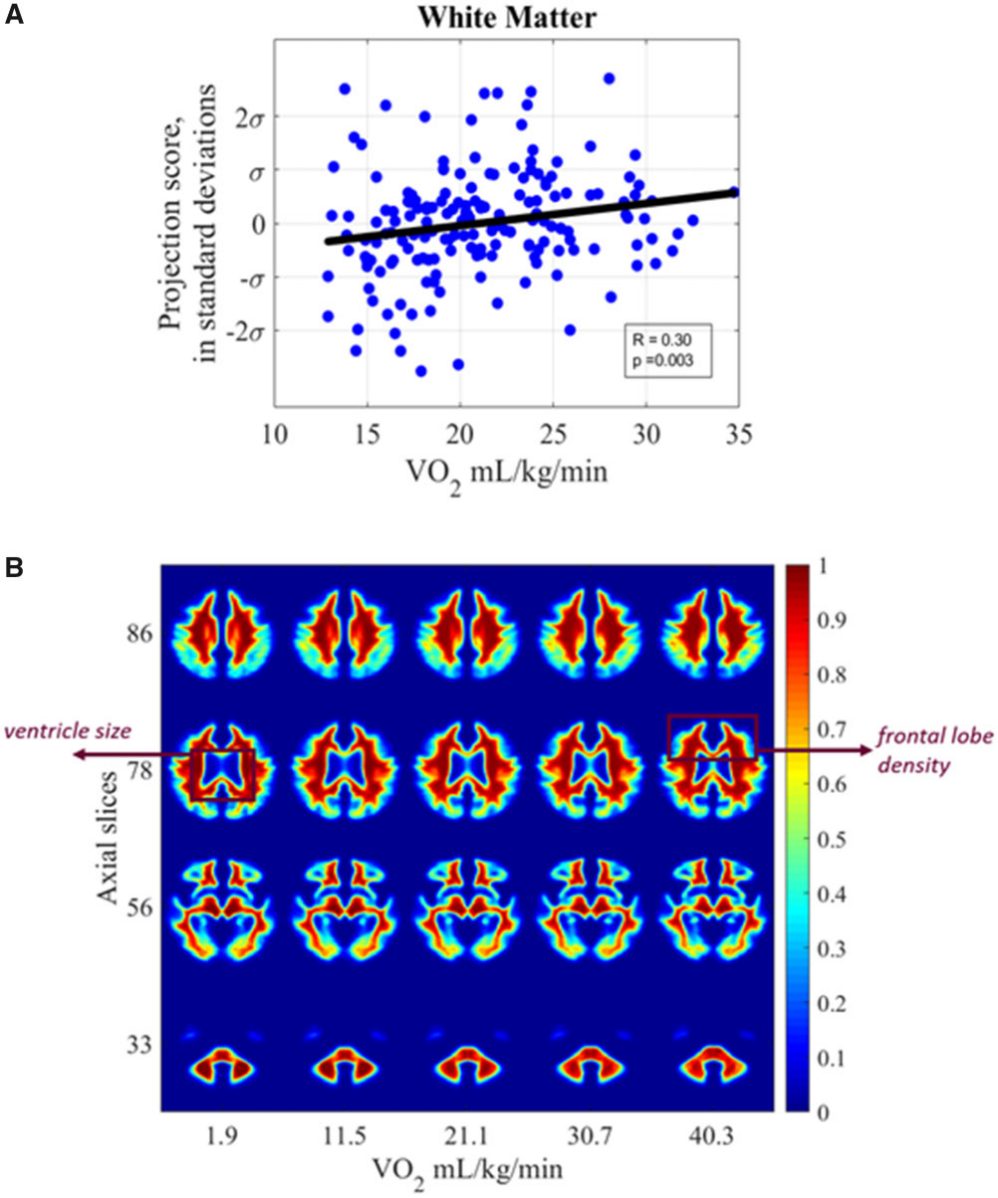
**White matter versus cardiorespiratory fitness.** (**A**) Scatter plot showing relationship between subject $VO_{2}$ peak and white matter distribution with extrapolated ages (**B**) synthetic images generated by transport-based morphometry directly visualizing the shifts in white matter distribution with $VO_{2}$ peak extrapolated from the study population. The images have been colorized for ease of interpretation. Scale bars correspond to the normalized intensity for each generated 3D image. Select interpolated images in the *z*-plane are shown that best summarize the differences.

Finally, the association between WM and CRF as measured in terms of $VO_{2}\;\mathrm{peak}$ was significant in the transport domain after correcting for covariates of age, gender, and level of education (Pearson’s *r* = 0.30 and *P* = 0.003) in Fig. 7A. The images in Fig. 7B are computer-generated by inverse TBM transformation to visualize the shifts discovered through automated TBM analysis. Tissue shifts across varying CRF levels were extrapolated based on the generative TBM model. We observe WM density associations in Fig. 7B such that higher CRF is associated with less peri-ventricular WM loss. In slice 78, we see mild WM density shifts in the frontal lobe regions. Again, the pattern of morphologic associations appears to be opposite those discovered using TBM between WM and ageing, described further in Localizing regions of dependent tissue displacement. There is a 0.58 mm displacement in WM tissue for every 10 ml/kg/min decrease in CRF.

### Interaction between cardiorespiratory fitness and age

#### Overlap of main effects

As the characteristic tissue displacements with age and with $VO_{2}$ peak occur in overlapping brain regions, the linear dependence between the respective patterns were measured. Table 3 summarizes the correlation between the pattern of GM and WM tissue displacement due to a 10-year increase in age and 10 ml/kg/min decrease in $VO_{2}$ peak. The pattern of GM and WM tissue displacement with lower CRF was found to overlap significantly with that seen in healthy ageing, as further elucidated in Table 4. Correlation across each region of interest was determined correcting for multiple comparisons. The variance explained in GM tissue is 55%, whereas that in WM tissue is 38%. Regional localization of these effects is summarized in Table 4. A linear regression model fitted with tissue shifts with age versus CRF suggest that a 10-year increase in age correlates to a 3.6 ml/kg/min decrease in $VO_{2}$ peak for WM tissues but 5.9 ml/kg/min decrease in $VO_{2}$ peak for GM tissues.

**Table 3:** Tissue redistribution due to a 10-year increase in age versus 10 ml/kg/min decrease in $VO_{2}$ peak

Tissue	Pearson’s correlation coefficient	*P*
Grey matter	0.74	<0.001
White matter	0.62	<0.001

Pearson’s correlation coefficient is computed to assess whether the tissue displacements in transport space due to age and CRF have a linearly dependent relationship.

**Table 4:** Tissue displacement for a 10-year age increase versus 10 ml/kg/min decrease in CRF across regions of interest

Region	Ageing (%)	Fitness (%)	Pearson correlation coefficient	*P*-value
Precentral gyrus	3.85	3.75	0.15	<0.0001
Superior frontal gyrus (dorsolateral)	3.85	4.57	0.37	<0.0001
Superior frontal gyrus (orbital part)	0.61	1.14	0.51	<0.0001
Middle frontal gyrus	3.54	6.81	0.12	<0.0001
Middle frontal gyrus (orbital part)	0.51	1.03	0.44	<0.0001
Inferior frontal gyrus (opercular part)	1.05	1.42	0.34	<0.0001
Inferior frontal gyrus (triangular part)	2.19	2.44	0.32	<0.0001
Inferior frontal gyrus (orbital part)	1.36	1.83	0.26	<0.0001
Rolandic operculum	1.02	1.39	0.30	<0.0001
Supplementary motor area	3.73	1.63	0.32	<0.0001
Olfactory cortex	0.25	0.34	0.09	<0.0001
Superior frontal gyrus (medial)	2.70	2.72	0.44	<0.0001
Superior frontal gyrus (medial orbital)	0.49	0.88	0.44	<0.0001
Rectus gyrus	0.40	0.76	−0.09	<0.0001
Insula	1.10	2.44	0.31	<0.0001
Anterior cingulate and paracingulate gyri	1.18	1.48	0.22	<0.0001
Median cingulate and paracingulate gyri	3.06	1.70	0.39	<0.0001
Posterior cingulate gyrus	0.59	0.46	0.80	<0.0001
Hippocampus	1.43	1.17	0.55	<0.0001
Parahippocampal gyrus	1.72	1.25	0.62	<0.0001
Amygdala	0.26	0.26	0.36	<0.0001
Calcarine fissure and surrounding cortex	3.72	3.30	0.92	<0.0001
Cuneus	2.20	2.22	0.85	<0.0001
Lingual gyrus	3.98	3.37	0.92	<0.0001
Superior occipital gyrus	2.01	2.04	0.79	<0.0001
Middle occipital gyrus	4.46	4.10	0.70	<0.0001
Inferior occipital gyrus	1.93	1.53	0.89	<0.0001
Fusiform gyrus	3.70	2.92	0.68	<0.0001
Postcentral gyrus	3.98	3.95	0.22	<0.0001
Superior parietal gyrus	2.32	2.35	0.63	<0.0001
Inferior parietal, but supramarginal and angular gyri	1.73	2.44	0.15	<0.0001
Supramarginal gyrus	1.62	2.08	0.28	<0.0001
Angular gyrus	1.58	1.93	0.44	<0.0001
Precuneus	4.00	3.65	0.69	<0.0001
Paracentral lobule	1.57	0.78	0.58	<0.0001
Caudate nucleus	1.87	1.48	0.72	<0.0001
Lenticular nucleus, putamen	0.65	1.29	0.005	0.28
Lenticular nucleus, pallidum	0.20	0.32	0.45	<0.0001
Thalamus	1.55	1.42	0.80	<0.0001
Heschl gyrus	0.26	0.34	0.38	<0.0001
Superior temporal gyrus	4.41	4.39	0.49	<0.0001
Temporal pole: superior temporal gyrus	2.16	2.06	0.60	<0.0001
Middle temporal gyrus	8.34	7.08	0.46	<0.0001
Temporal pole: middle temporal gyrus	1.43	1.40	0.71	<0.0001
Inferior temporal gyrus	5.44	4.08	0.62	<0.0001

Among the tissue shifts in AAL regions-of-interest, the percentage of tissue displacement localizing to each region is reported over a 10-year increase in age or 10-pt drop in CRF. Statistical significance was determined after correcting for multiple comparisons. AAL = automated anatomic labelling.

#### Summary of age × cardiorespiratory fitness interaction

When assessing the age × CRF interaction, CRF was found to weakly modify the relationship between age and GM tissue distribution (Pearson’s *r* = 0.23, *P* = 0.02) when correcting for gender and level of education and removing the partial effect of age from CRF. The variance explained (*R*^2^) was 5%. However, CRF did not significantly modify the relationship between age and WM tissue distribution (Pearson’s *r* = 0.22, *P* = 0.08).

### Localizing regions of dependent tissue displacement

The average redistribution in tissue density was computed for each of the 90 bilateral regions segmented by the automated anatomic labelling probabilistic structural atlas.^28^ Table 4 summarizes the mean tissue displacement either every 10-year increase in age or every 10 ml/kg/min decrease in $VO_{2}$ peak across each region. With ageing, the areas localizing the greatest tissue shifts were the middle temporal gyrus, inferior temporal gyrus, middle occipital gyrus, superior temporal gyrus, precuneus, postcentral gyrus, lingual gyrus, precentral gyrus, superior frontal gyrus (dorsolateral). Areas of least tissue shift with ageing included the amygdala, pallidum, superior frontal gyrus (orbital part), olfactory cortex and cingulate.

The regions with the greatest tissue shifts with CRF were the middle temporal gyrus, middle frontal gyrus, superior frontal gyrus (dorsolateral), superior temporal gyrus, middle occipital gyrus, inferior temporal gyrus, postcentral gyrus, precentral gyrus and lingual gyrus. Similarly, the areas with least tissue shift with CRF were the frontal gyrus (orbital part), amygdala, pallidum, cingulate and olfactory cortex.

Interestingly, the areas with the highest correlation between age- and CRF-dependent tissue shift were the thalamus, cingulate, caudate, middle temporal gyrus, occipital gyrus, calcarine cortex, cuneus and lingual gyrus. The regions with lowest correlation between age- and CRF-dependent tissue shift were rectus gyrus, olfactory cortex, middle frontal gyrus, precentral gyrus, inferior parietal (supramarginal and angular gyri), anterior cingulate and paracingulate gyri and postcentral gyrus. All regions had a statistically significant correlation between age- and CRF-dependent fractional tissue density changes.

Although our study reports that disproportionate shift in frontotemporal cortex and relative preservation in the basal ganglia occur with both ageing and CRF, the specific pattern of shift is not perfectly concordant between the two main effects, as seen in Table 4.

## Discussion

Mitigating ageing-related brain tissue decline is of particular interest for an ageing population. CRF is potentially neuroprotective, yet its influence on brain tissue is not fully characterized. This study performs a quantitative investigation of brain tissue distributional changes due to CRF vis-à-vis ageing using the TBM framework.^16^ First, we validated ageing-dependent microstructural shifts using TBM against known shifts from extant literature.^1^^,^^29^ Next, we investigated the phenotype of CRF-related tissue loss for the first time. We observed close quantitative overlap between tissue decline with ageing and decreasing CRF at a population level. We affirmed the study hypothesis that CRF is associated with disproportionate shifts in the frontotemporal regions, with relative preservation of the basal ganglia by visualizing biophysical tissue loss enabled by TBM. Tissue displacement may potentially be used clinically as it can measure variations in brain morphology in a lossless manner.

This research overcomes methodological limitations of previous studies by enabling direct examination of tissue distribution. Prior studies performed volumetry^5^^,^^9^^,^^10^^,^^17^^,^^30^^,^^31^ or voxelwise statistics.^11^^,^^14^ However, as Supplementary Fig. A1 demonstrates, brain parenchymal volume has little correlation with brain tissue distribution. As previous authors noted, volume may not be a sufficient descriptor of brain tissue preservation.^15^ Erickson et al.^4^ reported that structural perturbations in the striatum were not captured by volume change alone as a metric. Furthermore, VBM did not fully characterize the main effect of CRF on brain tissue density.^14^ In contrast, measuring brain tissue distribution using 3D TBM obviates the need for *a priori* features. Moreover, the transport space better characterized the underlying structure of the data when compared to voxel-based morphometry or the original image domain (see Supplementary material A4). The automated detection of tissue changes associated with CRF without human guidance is a significant advancement in this study.

Another key contribution of the 3D TBM technique is direct biophysical interpretation of the discovered phenotypic shifts (Figs. 6B and 7B). Traditional voxel-based methods look at voxelwise metrics which have no physical significance.^32^ To localize results or connections to particular areas, VBM methods use saliency maps or heat maps (see Supplementary material A4). As a result, this research fills a critical gap in understanding tissue redistribution in ageing and CRF.

Comparative assessment of ageing-related microstructure against CRF-related microstructure is another contribution of this study. We observed that main effects of ageing and CRF were not entirely overlapping, as variance explained in GM tissue was 55%. Fletcher et al.^15^ also reported discordant changes in certain ROI volumes between ageing and CRF. TBM discovers that frontotemporal regions were most affected by both ageing and CRF. Previous studies described accelerated age-related frontotemporal decline^33^ with relatively preserved limbic structures^34^ and relatively greater decline in hippocampus/parahippocampus.^35^ For CRF, previous studies also reported that frontal and prefrontal cortex volumes^9^^,^^14^ were disproportionately associated with CRF compared to other lobes.^30^ Although there is evidence that CRF is associated with brain areas involved in executive function and memory,^9^^,^^15^^,^^17^^,^^33^^,^^36^ association of CRF with other brain regions has received less attention. Finally, the dorsolateral frontal cortex, involved in executive function tasks such as planning and social behaviour,^33^ demonstrated relatively larger CRF shifts than other regions. However, the amygdala and orbital frontal cortex, commonly called the limbic frontal lobe,^33^ showed some of the smallest shifts with CRF compared to the dorsolateral part. These are novel findings with future clinical significance as we know of few other studies reporting these changes. Furthermore, these findings may have implications for frontotemporal dementia; an active lifestyle slowed clinical deterioration in adults with familial frontotemporal dementia.^37^

TBM reveals novel discordant shifts between ageing and CRF. Particularly, the middle frontal gyrus, inferior parietal (supramarginal and angular gyri), and anterior cingulate/paracingulate gyri all showed markedly greater shift with CRF than with ageing. Cingulate gyrus was associated with CRF in volumetric analysis.^10^ Given the areas and networks served by the anterior cingulate/paracingulate gyri, this research finds that CRF may potentially help maintain functions and behaviours in empathy^38^ and impulse control.^39^ CRF may also potentially help regulate networks and pathways supporting attentional control and semantic/number processing, memory retrieval, and cross-modal information integration through shifts in the angular gyri^40^ and middle frontal gyrus.^41^ Therefore, CRF may provide benefits beyond preserving brain regions involved in executive function that decline with age. These findings could potentially generalize to dementia-affected older adults. For example, in patients with mild cognitive impairment, facial emotional processing was impaired prior to cognitive deficits.^42^ Exercise intervention could possibly help preserve or reduce early emotional processing deficits during mild cognitive impairment—at a potentially reversible stage of dementia.

This study indicates that both CRF and ageing spare the basal ganglia, with robust overlap. Previous studies on the basal ganglia have reported inconsistent results. While a prior study found no significant relationship between putamen/globus pallidus volume and cardiovascular fitness in older adults,^43^ another study found significant association of the caudate nucleus, putamen, and globus pallidus volumes with performance in a Task Switching paradigm, mediated by CRF.^44^ Fletcher et al.^15^ reported a positive association between basal ganglia and CRF, but not with age. Our research suggests that ageing and CRF may influence motor and sensory pathways in different ways. We find that postcentral gyrus and precentral gyrus undergo relatively more discordant shifts between ageing and CRF, suggesting that CRF may potentially help maintain balance and mobility via more complex pathways.

Furthermore, as many neurodegenerative disorders such as Parkinson’s disease exhibit early basal ganglia changes,^45^ this research could potentially help identify early pathologic degeneration. CRF change is an independent risk factor in conversion to dementia^46^ and is modifiable through exercise intervention including in Parkinson’s disease.^47^^,^^48^

Animal studies using exercise training paradigms have largely informed mechanisms by which CRF interacts with the brain. First, exercise is linked to angiogenesis in the animal brain.^49^ In the human brain, blood flow velocity in the middle cerebral artery declines with age but increases with endurance-training.^50^ Other studies reported increased blood flow to the hippocampus following exercise intervention in humans^51^ and to anterior cingulate with exercise training.^52^ In addition to blood flow, a second potential mechanism is neurogenesis.^14^ In mice, exercise reversed hippocampal neurogenesis decline.^53^ Third, exercise may increase neural growth factor expression. In rodent studies, there were reported increases in both striatal-derived neurotrophic factor and neural activity in the striatum with exercise.^54^^,^^55^ Several studies report higher VO_2_ peak in humans associated with greater increases in brain-derived neurotrophic factor concentration following exercise.^17^^,^^56^

There were several limitations of this study. First, this was a cross-sectional study examining associations between CRF and brain tissue distribution in older adults. Longitudinal studies may further elucidate causality of the relationship between brain tissue distribution and CRF. Second, this research found that CRF weakly modifies the relationship between age and GM tissue distribution, accounting for 5% of the variance. Several studies demonstrated that cross-sectional associations between CRF, brain morphology, and function overlap, at least in part, with those that are found in interventions^4^^,^^5^^,^^8^^,^^9^^,^^14^^,^^44^. Thus, a future randomized clinical trial could further elucidate influence of CRF on the trajectory of age-related GM shifts. Third, we account for covariates of age, gender, levels of education, and total brain parenchymal volume (Supplementary material A1); there may potentially be other confounds as well. However, increasing the number of covariates comes with a trade-off of study power and multicollinearity.

CRF is mediated by a host of factors, including regularity of physical activity, genetics, smoking, and metabolic and cardiovascular comorbidities.^57^ Future longitudinal studies could help elucidate the role of exercise intervention in mitigating brain tissue losses in both normal ageing and neurodegenerative disorders, as it has been shown to increase CRF.^58^ Finally, the ability of 3D TBM as a new neuroinformatic technology to bridge structure-function associations in the brain in a fully automated manner may aid assessment of other therapeutic strategies beyond CRF to mitigate brain ageing and neurodegenerative diseases.

## Supplementary material

Supplementary material is available at *Brain Communications* online.

## Funding

This work was supported in part by National Science Foundation award CCF 1421502, and National Institutes of Health awards 710 R01 GM090033, GM130825, as well as the National Institute on Aging awards R01 AG25667, R01 AG25302 and National Institutes of Health awards P30 AG024827, P30 MH90333, R01 AG053952 and R01 DK095172.

## Competing interests

The authors report no competing interests.

## Supplementary Material

fcab228_Supplementary_Data

## Abbreviations

CRF =: cardiorespiratory fitness
GM =: grey matter
TBM =: 3D transport-based morphometry
WM =: white matter
