# Supplementary material for: Investigating impact of cardiorespiratory fitness in reducing brain tissue loss caused by ageing
Source: Brain Commun. 2021 Nov 18;3(4):fcab228. doi: 10.1093/braincomms/fcab228 (PMC8669566; doi:10.1093/braincomms/fcab228)
Supplement: fcab228_Supplementary_Data [file fcab228_Supplementary_Data.docx]

**Supplementary Material**

*A1. Investigating the influence of brain volume on brain tissue distribution*

In contrast to our approach focusing on brain tissue distribution, many previous reports have focused on a study of brain volume. Here, we investigate the relationship between brain tissue distribution and total brain parenchymal (TBP) volume. We perform a regression analysis in the transport domain where brain tissue distribution has been quantified. Correcting for covariates of age, gender, levels of education, there was not a significant association between tissue distribution pattern and TBP gray matter volume in the TBM analysis. The scatter plot in Supplementary Figure A1a demonstrates that there was not a significant correlation between TBP gray matter volume and brain tissue distribution.

Similarly, we quantify the relationship between TBP white matter volume and white matter distribution as measured in the transport domain. Although there was a correlation between TBP white matter volume and white matter tissue distribution, the variance explained (R^2^) was 8.4% in Supplementary Figure A1b.

Furthermore, the relationship between CRF and gray matter distribution remained significant when TBP gray matter volume was included as a covariate alongside age, gender, and levels of education (Pearson’s r = 0.38, p < 0.001). Similarly, the relationship between CRF and white matter distribution remained significant when TBP white matter volume was included as a covariate alongside age, gender, and levels of education (Pearson’s r = 0.32, p = 0.001).

Therefore, we confirmed that the study of brain tissue distribution assesses novel information not assessed by previous brain volumetry studies.

**(a)**

**(b)**

**Supplementary Figure A1.** Regression analysis. (a) Scatter plot showing relationship between gray matter volume and gray matter distribution in the transport space. (b) Scatter plot showing the relationship between white matter volume and white matter distribution in the transport space.

*A2. Adjustment for body mass index for tissue displacement with CRF*

When BMI is included as a covariate alongside age, gender, years of education, the relationship with tissue displacement remains statistically significant. The relationship between CRF and gray matter distribution (Pearson’s r = 0.44, p < 0.001) and white matter (Pearson’s r = 0.33, p = 0.001), are shown with TBM-generated images in Supplementary Figure A2. The images are similar to Supplementary Figures 6b and 7b in the main text.

**(a)**

**

**(b)**

**

**Supplementary Figure A2.** Adjusting for BMI as a covariate alongside age, gender, years of education. TBM-generated images generated directly visualizing the shifts in (a) gray matter and (b) white matter distribution, respectively, with $VO_{2}$ peak extrapolated from the study population. The images have been colorized for ease of interpretation. Scale bars correspond to the normalized intensity for each generated 3D image. Select interpolated images in the z-plane are shown that best summarize the differences.

*A3. 3D Transport-based morphometry*

**A3.1 Discovering phenotypic shifts in brain tissue distribution**

3D transport-based morphometry facilitates pattern recognition as nonlinear, spatially diffuse patterns in the image domain can map to simple linear distances in the transport domain (Park et al., 2017). Consequently, information can be represented more sparsely in the transport domain. Prior work demonstrates that linear classification and regression in the transport domain can capture spatially diffuse, regionally interdependent and nonlinear brain tissue in the image domain (Basu et al., 2014; Kundu et al., 2018; Wang et al., 2013; Kolouri et al., 2016). These spatially interdependent patterns are underestimated by voxelwise analysis (Kundu et al., 2018). Therefore, transport analysis greatly facilitates modern regression problems.

A linearized version of the optimal transport (OT) metric is used in the transport domain, enabling the Euclidean distances in linear pattern analysis techniques (i.e. principal components analysis, regression etc.) to be utilized in the transport domain to discover complex patterns in an automated manner.

**A3.2 Visualizing novel phenotypic shifts**

A key contribution of TBM is that it is *generative*. Under a set of constraints satisfied by our 3D TBM analysis, the transport map is unique for a pair of images (Brenier, 1991; Kundu et al*.,* 2018). Therefore, through inverse transformation, any arbitrary new point interrogating in the transport domain (with respect to the common reference) can be directly visualized as a new brain image.

A key advance of this work is that 3D TBM enables direct visualization of the tissue displacements driving correlation between morphology and CRF.

*A4. Comparison with voxel-based morphometry analysis*

**A4.1 Experimental methodology**

We compared the results of 3D TBM analysis with those obtained using a voxel-based morphometry approach. The SPM12 software was used to generate gray matter and white matter tissue density maps. The standard VBM8 toolbox (Gaser, 2017) was used to normalize the images using affine followed by non-linear registration, correct for bias-field inhomogeneity, segmentation, and smoothing to create gray matter and white matter statistical parametric maps (Ashburner et al., 2005) modulated by the local volume expansion/contraction. Modulation helps to account for absolute differences in brain size.

The statistical parametric maps were processed separately, as for the GM and WM transport maps. The VBM maps were smoothed with the same Gaussian filter as applied for the transport maps. Next, they were concatenated and dimensionality-reduced following the procedure for the transport maps to enable direct comparison between transport-based analysis and voxel-based analysis. Similarly, the components comprising 90% of the variance were retained. The same regression analysis code was run on the VBM maps as for 3D TBM. The direction in the image space was sought among the set of VBM maps that maximized the correlation with either age, or CRF when correcting for covariates (age, gender, levels of education). Based on the computed direction in the VBM space with either 10-year increase in age or 10 VO_2_ mg/kg/min decrease in CRF, we generated dynamic heat maps for the voxels whose modulated density value changed more than 2 standard deviations above or below the mean image.

**A4.2 Principal components**

Comparing the number of principal components needed to capture the variance in the dataset, 90% of the variance is captured by 120 principal components for white matter VBM maps and 125 principal components for gray matter VBM maps. In contrast, in the transport domain, 90% of the variance is described by 16 gray matter components and 32 white matter components. In the original image domain, 90% of the variance is described by 131 gray matter components and 128 white matter components. Therefore, the transport domain components best characterize the underlying structure of the data, with fewest needed number of components. VBM better captures the structure of the dataset compared to the original image domain but requires more components to describe the same variation when compared to TBM.

The dimension of the gray matter map in the transport domain was 155 x 196 x 154 x 3 (corresponding to 1.4 x ${10}^{7}$elements in the transport map) and, in the voxel-based domain was 155 x196 x 154. The dimension of the white matter map in the transport domain was 135 x 176 x 134 x 3 (corresponding to 9.6 x ${10}^{6}$ elements in the transport map) and, in the voxel-based domain was 135 x 176 x 134. The differences in size result from post-processing.

**(b)**


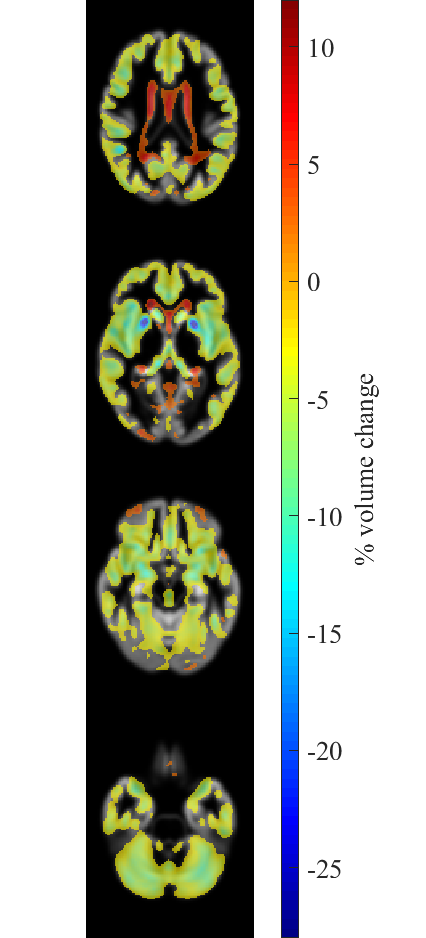


54

62

72

86

Axial slice

**10-yr change**

**A4.3 Age-related gray matter distribution**

For comparison, we repeat the analysis reported in the paper using gray matter density maps generated using the standard voxel-based morphometry pipeline. In Supplementary Figure A3a, analyzing the pixels on a fixed grid after warping results in a partial correlation that is not statistically significant between gray matter tissue distribution and age (Pearson’s r = 0.64, p = 0.054). Supplementary Figure A3b illustrates dynamic heat maps of gray matter images corresponding a 10 year decrease in age based on the most correlated direction computed using the voxel-based morphometry pipeline. First, we observe that although the VBM approach identifies many gray matter voxels correlated with age, it is not statistically significant. Second, the VBM approach measures a linear increase or decrease in individual voxel intensity (located on a fixed grid) with age. In contrast, TBM measures nonlinear shifts in the overall voxel distribution. In comparison to VBM, TBM discovers a direction that is statistically significant. The non-uniform shifts in brain tissue re-distribution with age are more visually apparent through inverse TBM transformation. We observe that measuring tissue distribution has an advantage in measuring the disproportionate effects on frontotemporal regions not found in slice 54 or 83 in Supplementary Figure A3b. In contrast, VBM demonstrates generalized decrease in gray matter density with aging, but less sensitive to nonlinear shifts. Finally, as TBM is generative, the images shown can describe physical shifts in brain tissue with age beyond dynamic heat maps compared to VBM.

**Supplementary Figure A3.** (a) Scatter plot showing relationship between subject age and gray matter when analyzed by VBM (b) heat map illustrating the percentage of volume change by voxel-based morphometry with 10 year decrease in age.

**A4.4 Age-related white matter distribution**

When the voxel-based morphometry technique was used to examine the partial relationship between white matter and age based on pixels on a fixed grid, the relationship was not statistically significant (Pearson’s r = 0.5, p = 0.234) as shown in Supplementary Figure A4a. Furthermore, Supplementary Figure A4b shows the dynamic heat maps in white matter tissue. In slices 61-86, the VBM approach demonstrates a loss of density from the frontotemporal regions. However, beyond voxel localization, the tissue density maps generated as part of VBM do not enable direct physical interpretation (Ashburner & Friston, 2004). Inverse TBM transformation enables direct physical understanding of how these localized regions change in appearance.

As the VBM approach assumes a fixed grid, nonlinear shifts in voxel distribution are better measured under the TBM approach. For example, Supplementary Figure 3b illustrates how TBM has captured the expected trends such as enlarging ventricles.

**(b)**


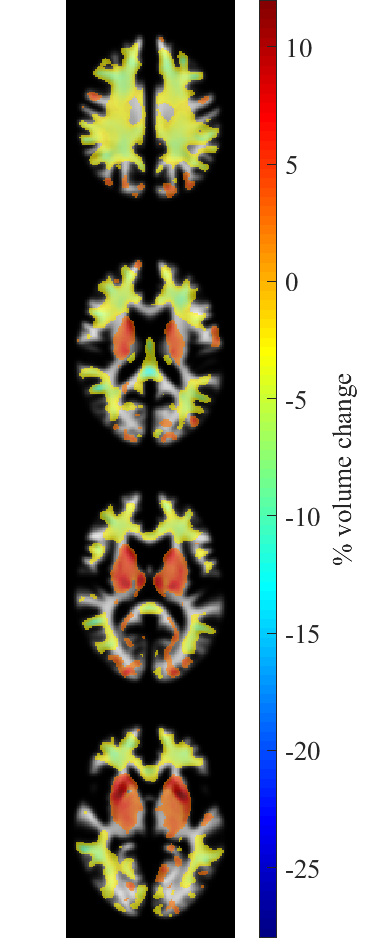


61

69

73

86

Axial slice

**10-yr change**

**A4.5 Cardiorespiratory fitness associations with gray matter tissue distribution**

In comparison with the TBM approach, the relationship between gray matter density and CRF assessed by voxel-based morphometry was not found to be statistically significant after correcting for covariates of age, gender, and levels of education (Pearson’s r = 0.55, p = 0.215) in Supplementary Figure A5a. Examining the images resulting from VBM in Supplementary Figure A5b, certain voxels were tagged as having an increase in density as a function of CRF. These voxels were diffusely distributed throughout the image, localized to the frontal and temporal regions disproportionately as in slice 80 and 69. Furthermore, although many gray matter voxels are correlated with CRF, overall it is not statistically significance. Furthermore, the VBM technique does not demonstrate the physical tissue distribution underlying saliency regions in response to higher CRF.

**Supplementary Figure A4.** (a) Scatter plot showing relationship between subject age and white matter when analyzed by VBM (b) heat maps generated by voxel-based morphometry modeling changes in linear intensity with age. Select images in the z-plane are shown that best summarize the voxel differences. The images are colorized for ease of interpretation.

**(a)**

**A4.6 Cardiorespiratory fitness associations with white matter distributions**


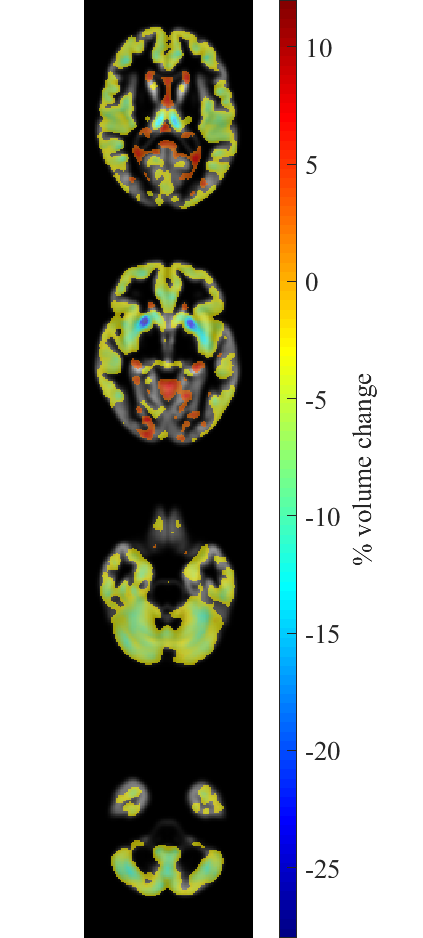


45

55

69

80

Axial slice

**10 mL/kg/min decline**

In comparison to TBM, the association between white matter density and CRF was not statistically significant when using VBM after correcting for covariates of age, gender, and levels of education (Pearson’s r = 0.36, and p = 0.472) in Supplementary Figure A6a. Furthermore, examining the images corresponding to the most correlated direction in Supplementary Figure A6b, nonlinear changes, most notably, ventricle size are not described through the heat maps. The VBM approach identifies which voxels on a fixed grid undergo linear increase or decrease in density with higher CRF, revealing that there was a diffuse density association with CRF level across multiple brain regions. However, unlike TBM, the VBM approach does not assess voxel movement with CRF, including how the heat map regions are mediated through changes in tissue re-distribution.

**Supplementary Figure A5.** (a) Scatter plot showing relationship between subject $VO_{2}$ peak and gray matter as assessed by VBM (b) images generated by voxel-based morphometry modeling gray matter intensity changes. Select interpolated images in the z-plane are shown that best summarize the changes in voxel intensity. The images are shown in terms of VBM density maps. The images are colorized for ease of interpretation.

**(a)**

**(b)**


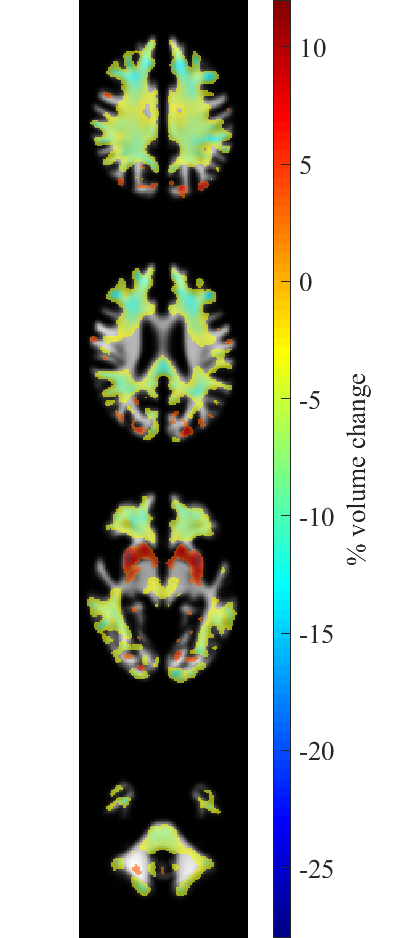


33

56

78

86

Axial slice

**10 mL/kg/min decline**

**Supplementary Figure A6.** (a) Scatter plot showing relationship between subject $VO_{2}$ peak and white matter as assessed by VBM (b) images generated by voxel-based morphometry modeling white matter intensity changes. Select interpolated images in the z-plane are shown that best summarize the morphology differences. The images are shown in terms of VBM density maps. The images are colorized for ease of interpretation.

*A5. Summary*

As prior studies have focused on either local regional volumetry or voxelwise analysis, this section investigated the relationship between tissue volume and brain tissue distribution and voxel-based morphometry regression. In Section A1, we observed that brain tissue distribution captures information largely uncorrelated from brain volume. In Section A4, VBM results were presented, an approach that assumes that voxels lie on a fixed grid after warping. The VBM analysis tagged voxels undergoing changes in tissue density with CRF. Both age and CRF were correlated with GM and WM voxelwise metrics but were not statistically significant under permutation testing. We observe that while VBM can describe associations with gross brain shape, it is not designed to describe the nonlinear movement of voxels with age or CRF that manifest as tissue texture. Furthermore, methods that rely on non-rigid registration such as VBM and DBM rely on accurate structural and functional alignment, (Bookstein et al*.,* 2001) a requirement that is difficult to achieve. However, in contrast to VBM, the 3D TBM approach can perfectly register gyri and sulci (Kundu et al., 2018) through a mass-preserving mapping. We observe with TBM how gray matter and white matter tissues re-distribute as a function of age or CRF. Measuring tissue re-distribution enabled TBM to measure relationships that were statistically significant, whereas the VBM approach did not.

Transport-based morphometry enables the best characterization of the data structure. In terms of principal components, 90% of the variance in gray matter distribution was captured with 16 principal components in the transport domain. In VBM analysis, 125 components were required to describe 90% of the variance, and in the original image domain, 131 components. We observe a similar trend for white matter distribution, which was described by only 32 components in the transport domain.

Finally, 3D TBM yields advantages in visualization as well. Voxelwise comparisons result in heat maps identifying which voxels are statistically significant; yet, a nonlinear model based on T1 spatial distribution in the transport domain can directly probe physical shifts in tissue organization. Under the VBM technique, considering each voxel as an independent variable did not capture the nature of interdependent and spatially diffuse changes compared to the 3D TBM approach. Unlike VBM, the TBM technique is *generative.* Although voxel-based morphometry indexes into voxels where metrics change with age or CRF, information related to the specific appearance of tissues is not recoverable because the changes in tissue texture and shape that are mediated by tissue re-distribution are lost. In assessing the relationship between tissue distribution and age, loss of matter from the frontotemporal regions and ventricular enlargement are well-described in the clinical literature (Brody, 1970). TBM discovered and visualized these shifts accurately, whereas VBM did not (Kundu et al., 2018). As TBM focuses on mass distribution of signal intensity, it is robust to absolute perturbations in signal intensity. TBM is advantageous as a discovery tool because information is not lost in the statistical analysis. This work demonstrates 3D TBM as a novel neuroinformatic approach for automated discovery and direct visualization of phenotypic shifts.

**References**

Ashburner J & Friston KJ (2005). Unified segmentation. *Neuroimage*, *26*(3), 839-851.

Friston KJ, & Ashburner J (2004). Generative and recognition models for neuroanatomy. *Neuroimage*, *23*(1), 21-24.

Gaser C (2017). “Structural Brain Mapping Group.” VBM at Structural Brain Mapping Group, University of Jena, <www.neuro.uni-jena.de/vbm/>.

Bookstein FL (2001). “Voxel-based morphometry” should not be used with imperfectly registered images. *Neuroimage 14*(6):1454-62.

Brody, H. (1970). Structural changes in the aging nervous system. In *The regulatory role of the nervous system in aging* (Vol. 7, pp. 9-21). Karger Publishers.

Kundu S, Kolouri S, Erickson KI, Kramer AF, McAuley E, Rohde GK (2018). Discovery and visualization of structural biomarkers from MRI using transport-based morphometry. *NeuroImage*, *167*, 256-275.

Park SR, Kolouri S, Kundu S, & Rohde GK (2017). The cumulative distribution transform and linear pattern classification. *Applied and Computational Harmonic Analysis*.
